# Cutting with sound or steel: A modern comparison of piezoelectric and rotary techniques in third molar surgery

**DOI:** 10.6026/973206300221895

**Published:** 2026-03-31

**Authors:** Nihalani Tanishq Shyamkumar, Paridhi Pateria, Shivani Adhikary, Ankita Sahare, Swati Bansal, Aditya Singh Tomar

**Affiliations:** 1 Department of Oral and Maxillofacial Surgery, Maharana Pratap College of Dentistry and Research Centre, Gwalior, Madhya Pradesh, India; 2 Department of Oral and Maxillofacial Surgery, District Hospital, Sagar, Madhya Pradesh, India

**Keywords:** Peizoelectric technique, conventional techniques, electricity driven drills, ultra surgery Peizoelectric unit, advances in oral and maxillofacial surgery, third molar impaction

## Abstract

Piezosurgery is a surgical technique that uses ultrasonic vibrations to precisely cut through bone tissue. It is commonly used in dental and maxillofacial procedures such as bone extraction, bone contouring and implant placement, providing benefits such as reduced soft tissue injury, decreased bleeding and improved patient comfort. This study has twenty six subjects and is a split mouth study in which bilateral third molar impactions are treated using piezosurgery and a traditional rotary approach. Piezosurgery has been shown to be a safe and effective osteotomy procedure that preserves soft tissue integrity while decreasing postoperative complications. Better postoperative recovery is taken into account to compensate for the extended operating time. Thus, we show that piezosurgery is more efficient and minimally invasive advancement for maxillofacial surgeons.

## Background:

The most common impacted teeth that need to be surgically removed are third molars. Caries and the emergence of related diseases are only two of the many issues that might be linked to partially or completely erupted teeth. One of the most crucial steps in surgical removal is cutting the bone, or osteotomy, for which a number of methods are employed. To split bone and tooth, chisels, osteotomes and mallets were employed. A quick method of removing enough bone was to use a rotating tool that rotates between 35,000 and 50,000 rpm. Because rotary cutting devices generate a high temperature during osteotomy, improper use may result in injury to the jaw bones [1]. This can hinder the healing and regeneration process and cause marginal osteonecrosis. Soft tissue injuries may arise from slippage. In oral and maxillofacial surgery, piezoelectric surgery was developed to address the drawbacks of the traditional rotational approach. Hippocrates most likely performed the first extraction ever recorded with an instrument known as a plumbean odontogagon. Wilfred Fish (1894-1974) developed the osteotomy technique. His contribution was the concept of sectioning the tooth with a chisel and mallet. This was accomplished with a "considerable blow" to the tooth [2]. Electricity-driven drills, which rotate between 22,000 and 25,000 rpm, were a quick and straightforward method of removing enough bone. They also offered a fair level of control over the quantity of bone removed. Pain, swelling, trismus and even paraesthesia of the lower lip or tongue are the most common post-surgical consequences. These might affect patient's quality of life on a social and biological level. A 2016 systematic review and meta-analysis by AL-Moraissi *et al.* [3] found that the piezoelectric surgical approach significantly reduces post-operative sequelae when utilised for third molar extractions the instrument's atraumatic and micrometric cutting action appears to be responsible for the low occurrence of post-operative complications [4]. Piezo surgery's micrometric cutting action necessitates a longer intervention time than using a bur, according to multiple studies, which could result in more postoperative discomfort and as an alternative to the mechanical and electrical tools used in traditional oral surgery, the peizoelectric device, also known as the piezosurgery device, was first created to cut bone atraumatically using ultrasonic vibrations [5].

## Methodology:

The current study was conducted in the Department of Oral and Maxillofacial Surgery at Maharana Pratap College of Dentistry and Research Center, Gwalior, Madhya Pradesh. After obtaining complete history, patients were examined clinically and were explained about the procedure, its complication and follow up period involved in the study. Informed consent was taken prior to the procedure.

## Inclusion criteria:

[1] Patient age 18 to 60 years.

[2] Patient either male or female.

[3] Surgical site is free of active infection.

[4] Patient is free of significant systemic disease.

## Exclusion criteria:

[1] Poor oral hygiene.

[2] Medically compromised patient.

[3] Pregnant or lactating women.

[4] HIV infection/HBS infection

[5] History of irradiation in head and neck

## Procedure:

All surgeries were done in conventional way and carried out by the same surgeon. Before the surgery, patients rinsed with 10% povidone- iodine mouthwash for 1 min. Inferior alveolar block and buccal anaesthesia were performed with 2 mL of 2% lignocaine HCl and 1:80000 epinephrine solution. A full-thickness envelope flap with a vertical releasing incision was reflected. In the control group, a conventional rotary handpiece and tungsten carbide burs were used under copious irrigation for removing the overlying bone (Figure 1 - see PDF). In the experimental group, piezosurgery (DTE Woodpecker Ultra surgery piezosurgical unit) was employed for the same purpose (Figure 2 - see PDF). Extraction wounds were closed with 3-0 silk sutures. The time passed from flap elevation untisuturing was recorded as "duration of the operation" (DO).

## According to group A:

[1] Obtaining written consent from patient included in the study.

[2] Patient will be operated under local anesthesia.

[3] Extraction of impacted or partially erupted third molar will be done using piezoelectric unit (Figure 3 - see PDF).

[4] Gently handle extraction site for clot formation and pack the wound site with gauze piece and post-operative instructions given to patient.

[5] Patient is medicated for 5 days and follow up done - 1 day, 7 days, 14 days, 1 month.

[6] While follow up radiographical assessment is done after 1 month to check the healing process.(EZDENT-I SOFTWARE VER.3.2)

## According to group B:

[1] Obtaining written consent from patient included in the study.

[2] Patient will be operated under local anesthesia.

[3] Extraction of impacted or partially erupted third molar will be done using conventional rotary technique (Figure 3 - see PDF).

[4] Gently handle extraction site for clot formation and pack the wound site with gauze piece and post-operative instructions given to patient.

[5] Patient is medicated for 5 days and follow up done - 1 day, 7 days, 14 days, 1 month.

[6] While follow up radio-graphical assessment is done after 1 month to check the healing process. (EZDENT-I SOFTWARE VER.3.2)

## Results:

Data were analysed using SPSS (Statistical Package for Social Sciences) 25.0 version. Data were analysed for probability distribution using Kolmogorov-smirnov test and were found to be normally distributed. Inter-group comparison of continuous variables was done using Independent t test. Intra-group comparison of continuous variables was done using Paired t test. Inter-group comparison of categorical variables was done using Marginal homogeneity test and Wilcoxon sign rank test. P-value<.05 was considered statistically significant. The mean ± standard deviation age of subjects was 26.92 ± 7.059 years. The majority of the subjects belonged to the age group of 18- 30 years (61.5%) (Table 1 - see PDF). The number of female subjects was greater than the number of male subjects (53.8% vs. 46.2%) (Table 2 - see PDF). The mean duration of operative procedure was significantly longer on Group A compared to Group B [36.61 ± 2.450 minutes vs. 26.69 ± 1.738 minutes] (p-value<.05) (Figure 4 - see PDF). At 1st follow up, the intensity of swelling was significantly greater in Group B compared to Group A with 57.7% and 0.0% of subjects with severe swelling in Group B And Group A respectively (p-value<.05). At 2nd follow up, most of the subjects in Group A no swelling (76.9%) whereas none of the subjects in Group B no swelling (0.0%). The intensity of swelling was significantly greater in Group B compared to Group A with 46.2% and 46.2% of subjects with mild and moderate swelling respectively in Group B and 0.0% and 23.1% of subjects with mild and moderate swelling respectively in Group A (p-value<.05) (Figure 5 - see PDF). At 1st follow up, the VAS score was significantly greater in group B compared to Group A (p-value <.05). At 2nd follow up, there was no significant difference in the VAS score between the groups (p-value>.05) (Figure 6 - see PDF). At 1st and 2nd follow up the mouth opening was significantly greater in Group A compared to Group B (p-value<.05) (Figure 7 - see PDF). Pre-operatively, there was no significant difference in MD width between the groups (p-value>.05). Post-operatively, the MD width was significantly greater in Group B compared to Group A (p-value<.05) (Figure 8 - see PDF).

## Discussion:

Piezosurgery has gained significant attention in recent years due to its ability to perform precise bone cutting while preserving soft tissue integrity. The present study aimed to compare the efficacy of piezosurgery with conventional rotary osteotomy in terms of operative time, postoperative pain, swelling, soft tissue healing, mouth opening and mesiodistal width changes. The results of this research indicate that while piezosurgery may take more time during the procedure, it provides considerable benefits by minimizing postoperative swelling, pain and trismus, in addition to improving soft tissue healing and maintaining mesiodistal width. These advantages render it an important alternative to traditional rotary osteotomy, especially in situations where precise bone preservation is necessary. Present study concludes showed that on average, piezosurgical patients (Group A) had a notably longer surgery time than those having a conventional rotary osteotomy (Group B). These results correspond with research carried out by Mantovani *et al.* [6] and Jiang's (2015) [7] noting that its micrometric cutting approach causes significant prolongation of operation, piezosurgery is greatly used. Still, the accuracy and lowered damage to surrounding tissues support its longer duration. The present study showed significantly reduced swelling in the piezosurgery group at both follow-ups compared to the conventional osteotomy group. These findings similar with the results of studies by Bennardo *et al.* [8] and Troedhan [9], who found a 50% reduction in swelling when piezosurgery was employed. The cavitation effect, which enhances haemostasis and reduces edema, is responsible for this reduced post-operative swelling. Pain was assessed using the Visual Analog Scale (VAS) at different follow-ups. At the first follow-up, pain scores were significantly lower in the piezosurgery group, whereas at the second follow-up, no significant difference was observed. Our findings are similar with those of Rullo *et al.* [10] who reported that piezosurgery results in reduced postoperative pain, due to its selective bone cutting and reduced trauma to surrounding tissues. Soft tissue healing was assessed at different follow-ups. Although no significant difference was noted between the groups at the first follow-up, by the second follow-up, both groups demonstrated similar healing pattern. These findings are as similar as Piersanti *et al.* [11] and De Melo Nogueira *et al.* [12] who reported that piezosurgery induces an earlier, increase in bone morphogenetic proteins (BMPs), thereby facilitating faster healing. Mouth opening was significantly better in the piezosurgery group at both follow-ups. Some literature also supports this finding, with studies by Goyal *et al.* [13] and Barone *et al.* [14] conclude that piezosurgery results in less trismus postoperatively. This can be because of the reduced muscular and ligamentous trauma caused by the micro vibration of piezosurgical instruments compared to conventional rotary osteotomy. Postoperatively the mesiodistal width was significantly greater in the conventional rotary osteotomy group. This concludes that piezosurgery allows for more controlled bone removal, preserving the anatomical structure. Same findings were reported by Arakji *et al.* [15] who found that piezosurgical cuts resemble those made by chisels rather than rotary burs, leading to improved bone preservation. Nihalani *et al.* [16] in 2025 conducted a split-mouth pilot study, in which piezosurgery showed a significantly longer operative time compared to the conventional rotary technique. However, it resulted in reduced early postoperative pain and swelling, with comparable soft tissue healing between groups. Overall, piezosurgery demonstrated superior early postoperative outcomes despite increased surgical duration.

## Conclusion:

Piezosurgery represents an advanced and minimally invasive modality in oral and maxillofacial surgery, enabling precise and selective osteotomy with minimal mechanical force, thereby significantly reducing the risk of injury to adjacent soft tissues and vital neurovascular structures, particularly in anatomically restricted areas. Although associated with a slightly longer operative time, its advantages including superior intraoperative visibility through continuous irrigation and enhanced illumination, reduced bleeding, and high precision in performing curvilinear osteotomies substantially improve surgical control and accuracy. Furthermore, piezosurgery contributes to improved postoperative outcomes by reducing edema and trismus, accelerating healing, preventing osteonecrosis, and promoting osteogenesis, thereby establishing it as a highly effective and biologically favourable technique for bone surgery and dental implantology.

## References

[1] Basheer SA (2017). J Contemp Dent Pract..

[2] Sifuentes-Cervantes JS (2021). Oral Surg Oral Med Oral Pathol Oral Radiol..

[3] Al-Moraissi EA (2016). Int J Oral Maxillofac Surg..

[4] Senan SEDA (2024). Univ J Pharm Res..

[5] Ravichandran V (2015). World J Adv Res Rev..

[6] Mantovani E (2014). J Oral Maxillofac Surg..

[7] Jiang Q (2015). Medicine (Baltimore)..

[8] Bennardo F (2023). Dent J (Basel)..

[9] Troedhan A (2018). J Oral Maxillofac Surg..

[10] Rullo R (2013). J Craniomaxillofac Surg..

[11] Piersanti L (2014). J Oral Maxillofac Surg..

[12] De Melo Nogueira DG (2023). J Oral Maxillofac Surg..

[13] Goyal M (2012). Br J Oral Maxillofac Surg..

[14] Barone A (2010). J Oral Maxillofac Surg..

[15] Arakji H (2016). Int J Dent..

[16] Nihalani T (2025). University Journal of Dental Sciences..

